# GOOD HEALTH AND WELL-BEING: ESSENTIAL TO ACHIEVING SDGS

**DOI:** 10.1093/geroni/igz038.295

**Published:** 2019-11-08

**Authors:** Hans Stohrer

**Affiliations:** Weill Cornell Medical University, New York, New York, United States

## Abstract

The importance of good health for older persons is a human right, good public policy, and practical. We argue that it is fundamental to achieving the UNs SDGs. Older people can be important assets in the achievement of SDGs but need to be in good health so as to be valuable, productive and contributing members of society. This includes functional health, cognitive health and capacity, mental health, sustainable long term care for non-communicable diseases, and quality palliative care provided in the home at the end of life. This means not only access to health care services and access to safe and effective medicines and vaccines, but also access to rehabilitation and physical therapy, mental health care, long term care, and hospice and palliative care. In conclusion, to achieve the United Nations SDG on Health, it is important to consider explicitly the health of older persons, including the public policy needs.

